# *In vitro* antibacterial effects of *Broussonetia papyrifera* leaf extract and its anti-colitis in DSS-treated mice

**DOI:** 10.3389/fcimb.2023.1255127

**Published:** 2023-10-16

**Authors:** Xiaoxiao Liang, Meng Ru, Zhenya Zhai, Jianzhen Huang, Wanwan Wang, Ruxia Wang, Zhihong Zhang, Kai-Min Niu, Xin Wu

**Affiliations:** ^1^ Institute of Biological Resources, Jiangxi Academy of Sciences, Nanchang, China; ^2^ College of Animal Science and Technology, Henan Agricultural University, Zhengzhou, China; ^3^ Henan Ground Biological Science & Technology Co., Ltd., Zhengzhou, China; ^4^ College of Animal Science and Technology, Jiangxi Agricultural University, Nanchang, China; ^5^ Tianjin Institute of Industrial Biotechnology, Chinese Academy of Sciences, Tianjin, China; ^6^ Institute of Subtropical Agriculture, Chinese Academy of Sciences, Changsha, China

**Keywords:** *Broussonetia papyrifera* leaf, antioxidant, metabolomic, microbiota, antibacterial

## Abstract

Recently, the hybrid *Broussonetia papyrifera* (BP) has been extensively cultivated and predominantly utilized in ruminants because of its high protein and bioactive compound content. In the present study, the effects of an ethanolic extract of BP leaves (BPE, 200 mg/kg) on mitigating 2% dextran sodium sulfate (DSS)-induced intestinal inflammation in mice were evaluated. BPE is rich in flavonoids, polyphenols, and polysaccharides, and displays potent antioxidant and antibacterial activities against pathogenic strains such as *Clostridium perfringens*, *Salmonella Typhimurium*, and *Salmonella enterica* subsp. *enterica in vitro*. In a mouse study, oral administration of DSS resulted in weight loss, incidence of diarrhea, enlargement of the liver and spleen, impaired colonic morphology, downregulation of both gene and protein expression related to intestinal antioxidant (Nrf2) and barrier function (ZO-1), decreased diversity of colonic microbiota, and 218 differentially altered colonic metabolites; however, co-treatment with BPE did not restore these modified aspects except for the liver index and colonic bacterial diversity. The singular treatment with BPE did not manifest evident side effects in normal mice but induced a mild occurrence of diarrhea and a notable alteration in the colonic metabolite profile. Moreover, a single BPE administration augmented the abundance of the commensal beneficial bacteria *Faecalibaculum* and *Akkermansia* genera. Overall, the extract of BP leaves did not demonstrate the anticipated effectiveness in alleviating DSS-induced intestinal inflammation.

## Introduction

*Broussonetia papyrifera* (BP) is a woody plant used in papermaking, traditional medicine, and pig feed in ancient China (Peng et al., 2019). It is predominantly distributed in the Southeast and East Asian countries. Characterized by a high content of digestible crude fiber, protein, and bioactive compounds, such as flavonoids, polyphenols, and polysaccharides, coupled with rapid growth, robust adaptability, and significant yield, BP is recognized as an economically, medicinally, and ecologically vital plant. It has been selected as one of China’s top ten targeted poverty alleviation projects (Hao et al., 2021). More recently, BP has been extensively cultivated to produce forage to address the shortage of Chinese feedstuffs. BP is mainly processed as silage using the entire plant for ruminants but is also partially utilized for pigs, laying hens, and carp to enhance production performance, meat quality, and immunity (Sheng et al., 2021; Tang et al., 2021; Wu et al., 2022; Zhang et al., 2022; Niu et al., 2023). The fermentation process can modulate bioactive compounds within BP, thereby enhancing its functionality and palatability (Du et al., 2022; Hao et al., 2022; Niu et al., 2022).

Raw BP also contains abundant bioactive compounds such as phenolic acids, flavonoids, and polysaccharides, which serve as foundational elements for antioxidant, anticancer, and anti-inflammatory effects (Tian et al., 2019; Cao et al., 2020). Papyriflavonol A, a flavonol compound isolated from the root bark of BP, exerts a fungicidal effect against *Candida albicans* (Sohn et al., 2010). Extracts of BP stem bark and wood exhibit elevated scavenging activities against 2,2-diphenyl-2-picrylhydrazyl hydrate (DPPH) radical, hydroxyl radical, and superoxide anion, and demonstrated reducing potential (Xu et al., 2010). Compounds isolated from root bark, such as flavanone, chalcone, dihydroflavonol, and 1,3-diphenylpropanes, display inhibitory effects on pro-inflammatory cytokine production in RAW264.7 cells (Ryu et al., 2019). Furthermore, polysaccharide extracts from BP fruits have high hydroxyl radical scavenging activity, ferric-reducing activity, and antibacterial properties against *Escherichia coli*, *Pseudomonas aeruginosa*, and *Staphylococcus aureus* (Han et al., 2016).

In the contemporary breeding industry, farm animals encounter challenges such as intensive density, environmental stress, feed contamination, and inclusion of new feed ingredients, all of which may induce intestinal inflammation (Zou et al., 2016; Meng et al., 2019). Bacterial infection also threatens intestinal health, further undermining the growth performance of farm animals (Gresse et al., 2017; Lopez-Colom et al., 2019). To date, there has been very limited information concerning the therapeutic or prophylactic effects of BP extracts on intestinal inflammation, despite the widespread application of BP silage in farm animals. Therefore, in this study, we aimed to investigate the impact of BPE on intestinal inflammation in a DSS-treated mouse model by evaluating the diarrhea status, intestinal morphology, colonic microbiota, and gene and protein expression associated with antioxidant, anti-inflammatory, and barrier functions.

## Materials and methods

### Ethics statement

Animal experiments were conducted in accordance with the procedures and guidelines set forth by the Animal Care and Use Committee of the Institute of Subtropical Agriculture, Chinese Academy of Sciences, and were approved under the designation ISA 2020-18.

### Preparation of *B. papyrifera* leaf extract

Dried *B. papyrifera* powder (0.5 kg), sourced from the local market, was soaked in 95% ethanol at a ratio of 1:10 (w/v) and stored at room temperature for one week. The supernatant was collected and filtered. This procedure was performed three times, and the supernatant was concentrated using a vacuum rotary evaporator (RE 2008, Shengye, Shanghai, China) paired with a cooling system (DLSB-10L-10, Yuhua, Hunan, China). The concentrated extract was then decolorized with petroleum ether and further concentrated by evaporation, culminating in freeze drying.

### Determination of bioactive compounds and total antioxidant capacity of BPE

The levels of total flavonoids, total polyphenols, total sugar, and total antioxidant capacity were determined following the protocols associated with the relevant kits obtained from Suzhou Keming Biotechnology Co., Ltd. (Suzhou, China). The active compounds within BPE were examined using liquid chromatography-tandem secondary mass spe ctrometry (LC–MS/MS). The instrument parameters, running conditions, and analysis protocols were aligned with our previously published methods (Zhai et al., 2021). An UltiMate 3000 (Thermo Fisher Scientific, Waltham, MA, USA) equipped with a C18 chromatographic column was used. Mobile phases A and B consisted of 100% acetonitrile and 0.1% formic acid and H_2_O and 0.1% formic acid, respectively. The elution gradient ran as follows: A: 0 min–7 min, 5%–50%; 7 min–8 min, 50%–75%; 8 min–9 min, 75%–80%; 9 min–11 min, 80%–90%; 11 min–15 min, 90%–95%; 15 min–20 min, 95%. The flow rate was maintained at 0.2 mL/min. MS/MS, equipped with Q-Exactive Focus (Thermo Fisher Scientific, Waltham, MA, USA), was configured as follows: ion source: ESI, atomization temperature: 300°C, sheath gas pressure: 40 ARB, auxiliary gas pressure: 10 ARB, transmission capillary temperature: 320°C, scanning mode: full scan, resolution 35,000, in-source CID: 0 ev, ddms2, resolution 17,500, HCD stepped NCE 10, 30, 50. The top 20 metabolites of BPE were identified.

### Determination of inhibitory effects of BPE

The inhibitory effects of BPE were determined according to a previously described method (Niu et al., 2019b). A total of six indicator pathogens implicated in animal intestinal diseases, including *E. coli* W25K, *E. coli* ATCC 35150, *E. coli* 8700 1.558, *Salmonella typhimurium*, *Salmonella enteritis* CMCC S007, and *Clostridium perfringens* ATCC 9120, were employed. Overnight cultures of these pathogens were prepared in LB (Luria–Bertani) medium. The final concentrations of BPE were adjusted to 25 mg/mL, 50 mg/mL, and 100 mg/mL in LB solution containing 1 × 10^6^ CFU/mL of the pathogens, and 1 mL of this solution was added to the wells of a 24-well plate (JET, Guangzhou, China). The plates were then incubated for 24 h at 37 °C. Samples were extracted at 0 h, 4 h, 6 h, 8 h, 12 h, and 24 h for viable cell number determination (Log_10_ CFU/mL) using an agar plating method. Distilled water served as a control, and the experiments were performed in triplicate.

### The mouse experiment and sampling

A total of 36 female C57BL/6 mice (20 g–25 g, aged 11 weeks) were acquired from the STJ Laboratory Animal Co., Ltd. (Hunan, China) for the purpose of this study. The mice were maintained under controlled conditions: temperature (22°C –25°C), humidity (55%–75%), with 12-hour light-dark cycles, and unrestricted access to both food and drinking water. Following a one-week acclimation period, the mice were randomly assigned to four distinct treatment groups: the control group (Con), BPE group (BPE), dextran sulfate sodium group (DSS), and DSS + BPE group (DSS + BPE). Each group contained three replicates, and each replicate consisted of three mice that were housed in a single cage.

For the mice in the Con and BPE groups, distilled drinking water was provided ad libitum, while the mice in the DSS and DSS + BPE groups were administered drinking water infused with 2% (w/v) DSS (36,000 M. Wt.–50,000 M. Wt., MP Biomedicals, Solon, OH, USA) over 7 days (**Figure 2A**). Concurrently, the mice in the BPE and DSS + BPE groups were subjected to oral gavage with 200 mg/kg body weight of BPE, whereas mice in the Con and DSS groups received an equivalent volume of PBS daily. The adopted dosage of BPE in the mouse experiment was described in an earlier study (Xu et al., 2022). All mice were subjected to weight assessment and daily evaluation of the diarrhea index. On Day 11, following three additional days of feeding, the mice were humanely sacrificed to facilitate sample collection. The sampling methodology was consistent with that described in an earlier study (Zhai et al., 2021). The mice were fasted for 6 h and were then euthanized by intraperitoneal injection of 2% pentobarbital sodium (45 mg/kg body weight). Blood samples were collected *via* enucleation of the eyeball. The distal ileum (near the cecum to 2 cm) and colon were collected and fixed in 4% neutral polyformaldehyde for intestinal histological analysis or frozen in liquid nitrogen for real-time quantitative PCR (RT-qPCR) analysis. Colonic contents were collected for the analysis of the intestinal microbiota.

### Histological morphology analysis

Intestinal histological alterations were evaluated by hematoxylin and eosin (H&E) staining. In brief, neutral polyformaldehyde-fixed ileum and colon samples were dehydrated using ethanol and xylene, followed by paraffin embedding. Paraffin-embedded tissues were then sectioned into 5 μm slices, deparaffinized, hydrated, and stained with H&E. Villus length (VL) and crypt depth (CD) were measured using an Olympus microscope paired with VistarImage software (Olympus, Japan) (Zhai et al., 2019).

### Histological immunofluorescence

The protocol for colon immunostaining was performed in accordance with previously established methods (Zhai et al., 2021). Following deparaffinization, rehydration, and serum blocking following antigen retrieval, the tissue sections were treated with primary antibodies. After overnight incubation at 4°C, the tissues were exposed to secondary antibodies and incubated in the dark at room temperature for 50 min. The slides were then treated with DAPI solution for 10 min and stored in the dark. A Nikon Eclipse C1 fluorescence microscope equipped with a Nikon DS-Us imaging system was used for image capture and analysis.

### Quantitative real-time (RT-PCR) analysis in colonic tissues

Quantitative real-time PCR (RT-qPCR) was performed to assess colon gene expression associated with antioxidant activity, inflammation, and epithelial barrier integrity following an established method (Zhai et al., 2021). The mRNA of colon samples was extracted using a columnar RNA extraction kit (Magen, Guangzhou, China), and the concentration was measured using a NanoDrop 2000C spectrophotometer (Thermo Fisher Scientific, Waltham, MA, USA). RNA was reverse-transcribed into cDNA using a commercial kit (CW2582M, CWBIO, Jiangsu, China). The primers used in this study were designed and synthesized by Shanghai Shenggong Biotechnology Co., LTD (Shanghai, China). The primer information is presented in **Supplementary Table 1**. RT-qPCR was conducted using a FastSYBR kit (CW0955M, CWBIO) following the manufacturer’s protocol and run in an ABI 7500 FAST system (Applied Biosystems Instruments, Thermo Fisher Scientific, United States) under standard running conditions. qRT-PCR was performed using the Ct value as the PCR threshold cycle number at the endpoint. The relative expression levels of target genes were normalized to the housekeeping gene glyceraldehyde 3-phosphate dehydrogenase (GADPH) using the ΔΔCT method (Ru et al., 2022).

### Colonic microbiota

Genomic DNA (gDNA) extraction, quality assessment, sequencing, and related analyses were performed in accordance with previously described methods (Niu et al., 2019a). Briefly, gDNA was extracted using the CTAB method (Niu et al., 2019a) and the concentration was quantified using a NanoDrop 2000. Purity and quality were determined using 1% agarose gel electrophoresis. The V4 hypervariable region of 16S rRNA was amplified using universal primers 515F and 806R. PCR and sequencing were performed on an Illumina NovaSeq 6000 platform using the commercial services of Norson Bioinformatics Co., Ltd. (Beijing, China). Sequence clusters exhibiting 97% similarity were identified as identical operational taxonomy units (OTU) utilizing UParse (version v 1.9.1). The Silva database (http://www.arb-silva.de/) with the Mothur algorithm was used to annotate the classification information of representative sequences. Alpha diversity indexes, such as Observed_species, Chao1, Simpson, and Shannon, were analyzed in QIIME (Version 1.7.0) and visualized using R software (Version 2.15.3). Principal component analysis at the OTU level was calculated in QIIME (Version 1.9.1).

### Untargeted colonic metabolomics

Untargeted metabolomic analyses of colonic samples were performed using an LC–MS/MS platform. Data were interpreted using the commercial services of Novogene Biotech Co., Ltd. (Beijing, China). Colonic sample preparation and LC–MS/MS analysis conditions were implemented in accordance with a previously described method (Lu et al., 2019). Metabolites with VIP >1.5, P-value <0.05, and fold change (FC) >2 were designated as differentially expressed metabolites (DEMs). The identified metabolites were functionally annotated using the Kyoto Encyclopedia of Genes and Genomes (KEGG) database and the annotated metabolites were mapped to the KEGG pathway database. The enrichment of metabolic pathways among the differential metabolites was subsequently performed.

### Statistical analysis

The data generated in the mouse study were subjected to statistical analysis by one-way analysis of variance (ANOVA) using SPSS (version 20, SPSS Inc., Chicago, IL, USA). Tukey’s HSD multiple range test was used to ascertain statistically significant disparities (P <0.05) in the mean responses to distinct treatments. Data are presented as the mean ± standard error (SEM) of the mean of triplicate groups. Notations such as * denotes P <0.05, ** represents P <0.01, and *** indicates P <0.001. GraphPad Prism 7 was used for figure creation.

## Results

### Antioxidant activity and bioactive compound profile of BPE

The total contents of flavonoids, polyphenols, and sugars in BPE were quantified as 67.72 mg/g, 343.62 mg/g, and 181.81 mg/g, respectively. The scavenging activity against superoxide radicals was 45.53%, and the principal bioactive compounds are detailed in **Table 1**. Within BPE, the six predominant bioactive compounds, quinic acid, geniposidic acid, 1-caffeoyiquinic acid, cryptochlorogenic acid, caffeic acid, and citric acid, each constituted more than 1%.

**Table 1:** The main active compounds identified in BPE.

#	Compounds	Relative abundance (%)
1	D-(−)-Quinic acid	4.91
2	Geniposidic acid	3.78
3	1-Caffeoyiquinic acid	2.09
4	Cryptochlorogenic acid	1.66
5	Caffeic acid	1.24
6	Citric acid	1.12
7	Gluconic acid	0.68
8	β-D-Glucopyranuronic acid	0.63
9	L-(−)-Malic acid	0.57
10	Baicalin	0.39
11	Cynaroside	0.31
12	Trans-Aconitic acid	0.30
13	1.3-Dicaffeoylquinic acid	0.25
14	Quercetin-3β-D-glucoside	0.20
15	2.3-Dihydroxybenzoic acid	0.19
16	Emodin	0.16
17	Rubiadin	0.12
18	4-Hydroxybenzoic acid	0.12
19	Adenine	0.11
20	Esculetin	0.09

### Inhibitory activity of BPE against intestinal pathogens

**Figure 1:**
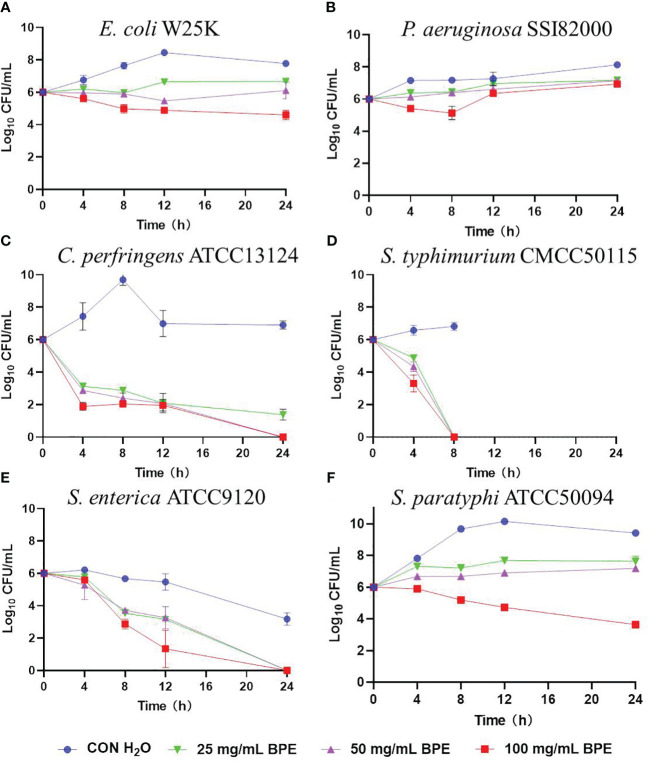
Inhibitory activity of BPE against intestinal pathogens. **(A)** Enterotoxigenic *Escherichia coli* W25K; **(B)** *Pseudomonas aeruginosa* SSI 82000; **(C)** *Clostridium perfringens* ATCC 13124; **(D)** *Salmonella typhimurium* CMCC 50115; **(E)** *Salmonella enterica* subsp. enterica ATCC 9120; and **(F)** *Salmonella paratyphi*-B ATCC 50094.

The *in vitro* inhibitory activities of BPE against several prevalent intestinal pathogens were evaluated (**Figure 1**). Three different concentrations of BPE were found effective in impeding the growth of *C. perfringens* ATCC 13124 (**Figure 1C**), *S. typhimurium* CMCC 50115 (**Figure 1D**), and *S. enterica* subsp. enterica ATCC 9120 (**Figure 1E**), without affecting the growth of enterotoxigenic *E. coli* W25K (**Figure 1A**), and *P. aeruginosa* SSI 82000 (**Figure 1B**). Only the highest concentration of BPE (100 mg/mL) demonstrated an antagonistic effect on the growth of *Salmonella paratyphi* B ATCC 50094 (**Figure 1F**).

### Effect of BPE on diarrhea, body weight, organ index, and colon length in normal and the DSS-treated mice

**Figure 2:**
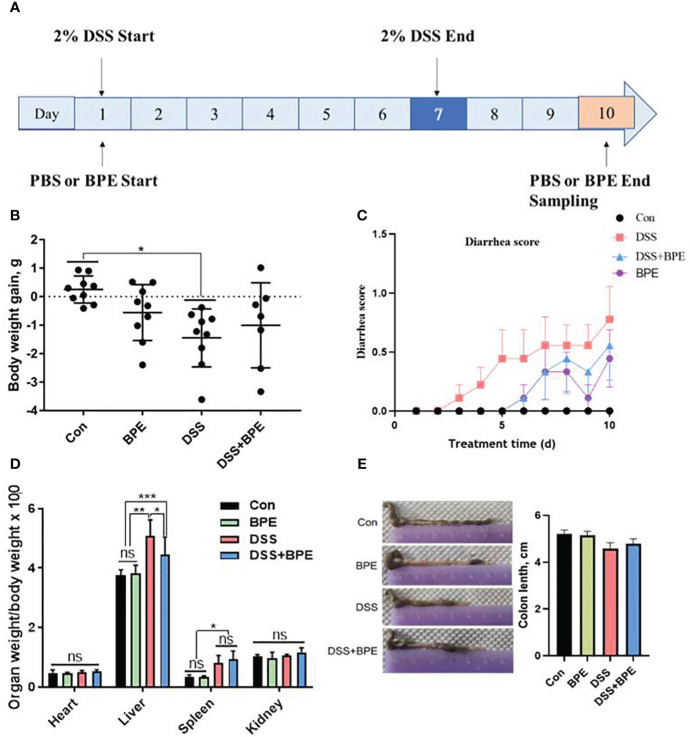
Effect of BPE on diarrhea, body weight, organ index, and colon length of DSS-treated mice. **(A)** The workflow of the experiment; **(B)** Body weight; **(C)** Diarrhea score; **(D)** organ index; and **(E)** Colon length. N = 9 mice/group, *p <0.05; **p <0.01; ***p <0.001. ns, no significant difference.

A mouse model of colitis was established by administering 2% DSS in drinking water over a 7-day period (**Figure 2A**). The effect of BPE on both normal (PBS gavage) and DSS-treated mice was explored. Compared to the control (Con) treatment, DSS treatment led to a marked reduction in body weight (**Figure 2B**), augmentation of liver and spleen indices (**Figure 2D**), and rapid onset of diarrhea (**Figure 2C**). While co-treatment with BPE did not counteract weight loss, it moderately mitigated diarrhea severity and liver weight gain compared with DSS treatment alone. The singular BPE treatment did not cause a notable change in body weight or organ index but induced slight diarrhea after 5 days of gavage. Colon length did not differ significantly among the four treatments (**Figure 2E**).

### Effect of BPE on intestinal morphology in normal and the DSS-treated mice

**Figure 3:**
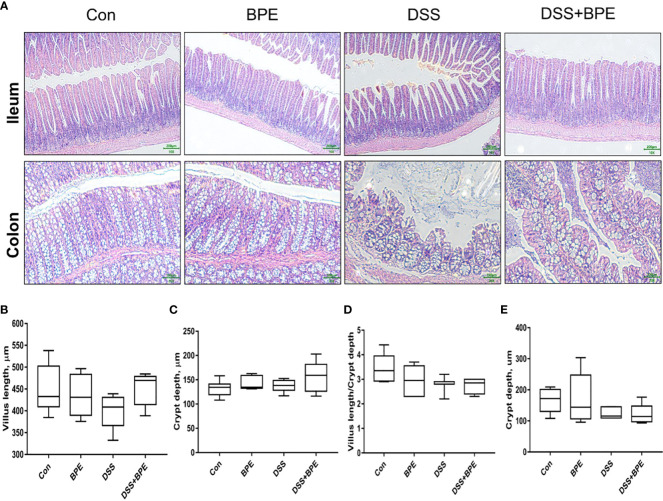
Effect of BPE on histomorphology of mice treated with DSS. **(A)** Representative photos of ileum and colon; Villus height **(B)**; Crypt depth **(C)**; Villus height/crypt depth ratio **(D)** in ileum; and **(E)** Crypt depth in colon, n = 6 mice/group.

The effect of BPE on the intestinal morphology of both normal and DSS-treated mice was examined (**Figure 3**). DSS treatment severely destroyed the colonic structure, which BPE co-treatment did not ameliorate by BPE cotreatment. Isolated BPE treatment caused minor damage to the colonic structure. In terms of ileal structure, no conspicuous differences were observed among the four treatments (**Figure 3A**). From numerical statistics, neither single nor co-BPE treatment led to significant alterations in villus length (VL), crypt depth (CD), and VL/CD ratio within the ileum and colon in comparison with the Con and DSS treatments.

### Effect of BPE on colonic gene expression involving antioxidant, inflammation, and barrier function in normal and the DSS-treated mice

**Figure 4:**
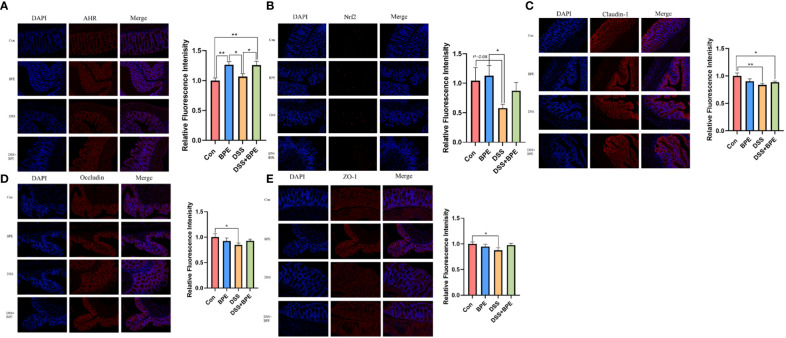
Effect of BPE on colonic protein expression related to antioxidant and barrier function. Representative immunofluorescent photos of **(A)** AHR; **(B)** Nrf2; **(C)** Claudin-1; **(D)** Occludin; and **(E)** ZO-1. n = 6, *p <0.05; **p <0.01.

We investigated the effects of BPE on colonic gene expression related to antioxidant activity, inflammation, and barrier function (**Table 2**). DSS treatment markedly downregulated the relative gene expression of CYP1A1, Nrf2, and TLR4, yet co-treatment with BPE did not suppress the expression of these genes when compared with Con treatment. A single BPE treatment did not also cause significant changes in any of the tested genes. Furthermore, the impact of BPE on the expression of proteins associated with antioxidant and barrier functions was assessed using colon immunofluorescence (**Figure 4**). In comparison to Con treatment, DSS treatment significantly reduced the protein expression of Nrf2, claudin-1, occludin, and ZO-1, and BPE co-treatment did not restore these levels. Treatment with isolated BPE did not induce any notable differences in these proteins. For AHR protein expression, a single BPE treatment increased its expression, as did co-treatment with BPE and DSS. These results demonstrate that BPE was not efficacious in rectifying DSS-induced dysfunctions related to intestinal antioxidant and barrier functionalities at both the gene and protein expression levels.

**Table 2:** Effects of BPE on colonic gene expression related to antioxidants, inflammation, and tight junction proteins.

Name	Treatment				*P*-value
	Con	BPE	DSS	DSS + BPE	
AHR	1.38 ± 0.40	1.05 ± 0.18	1.69 ± 0.36	2.53 ± 0.75	0.150
CYP1A1	1.63 ± 0.31a	1.39 ± 0.21a	0.01 ± 0.01b	0.02 ± 0.00b	0.000
Nrf2	2.21 ± 0.34a	1.56 ± 0.07ab	0.20 ± 0.05c	0.76 ± 0.22bc	0.000
ZO-1	1.61 ± 0.23	1.29 ± 0.18	1.17 ± 0.17	1.82 ± 0.40	0.308
TLR4	1.26 ± 0.18a	0.95 ± 0.03ab	0.52 ± 0.11b	0.68 ± 0.12b	0.006
IL-6	1.77 ± 0.31	1.61 ± 0.27	1.16 ± 0.47	0.82 ± 0.27	0.230
IL-10	1.30 ± 0.28	1.14 ± 0.18	1.06 ± 0.21	1.46 ± 0.35	0.720

### Effect of BPE on colonic microbiota in normal and the DSS-treated mice

**Figure 5:**
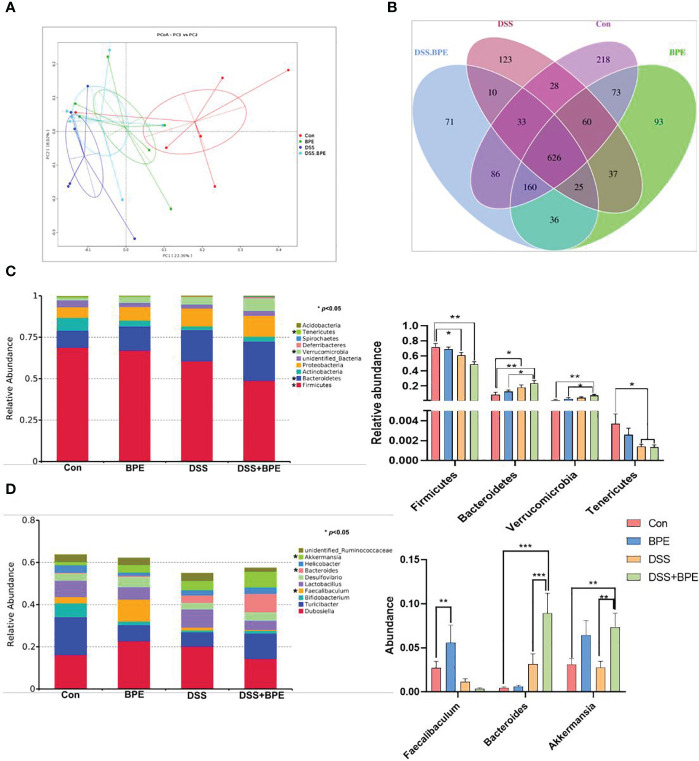
Effect of BPE on β-diversity of colonic microbiota and colonic microbial composition. **(A)** PcoA plot; **(B)** Venn diagram; **(C)** Relative abundance of dominant phyla; and **(D)** Relative abundance of dominant genera. n = 6, *p <0.05; **p <0.01; ***p <0.001.

The 16S rDNA sequencing methodology was used to assess the influence of BPE on the colonic microbiota. In comparison with the control (Con) treatment, there was a significant reduction in ACE- and Chao1-based α-diversity of the colonic microbiota in mice treated with DSS. This effect was reversed by BPE co-treatment; however, individual administration of BPE did not induce notable differences in α-diversity indices (**Table 3**). Regarding the similarity of colonic microbiota among the four treatments, the PCoA plot illustrated a distance order of DSS + BPE > DSS > BPE when compared with the Con treatment (**Figure 5A**). A Venn diagram displayed 626 core OTUs among the four treatments, with 218, 93, 123, and 71 unique OTUs identified in the Con, BPE, DS S, and BPE + DSS groups, respectively (**Figure 5B**). The colonic bacterial composition was profiled at both phylum (**Figure 5C**) and genus (**Figure 5D**) levels. At the phylum level, Firmicutes, Bacteroidetes, Actinobacteria, and Proteobacteria were the predominant phyla across all treatments. In comparison with Con treatment, DSS induced a significant decrease in the abundance of Firmicutes and Tenericutes, accompanied by an increase in Bacteroidetes abundance. Conversely, BPE supplementation in DSS-treated mice did not affect the altered phyla. A single BPE treatment did not alter the dominant phyla relative to those in the Con treatment. At the genus level, *Dubosiella* and *Turicibacter* were the most dominant genera among the four treatments. Compared with the Con treatment, DSS treatment resulted in a significant surge in Bacteroides abundance, an effect that was not restored by BPE co-treatment. Individual BPE treatments notably increased *Faecalibaculum* abundance, but co-treatment with BPE decreased it. Relative to the Con and DSS groups, BPE addition significantly augmented the abundance of *Akkermansia*.

**Table 3:** Effect of BPE on α-diversity of colonic microbiota in mice treated with DSS.

Indexes	Treatments				*P*-value
	Con	BPE	DSS	DSS + BPE	
ACE	647.6 ± 57.4a	571.6 ± 37.4a	467.0 ± 44.3b	528.9 ± 46.6a	0.080
Chao1	644.6 ± 57.6a	569.6 ± 36.6a	467.6 ± 45.3b	523.6 ± 46.1a	0.086
Shannon	5.4 ± 0.5	5.3 ± 0.1	5.4 ± 0.2	5.3 ± 0.1	0.996
Simpson	0.9 ± 0.0	0.9 ± 0.0	0.9 ± 0.0	0.9 ± 0.0	0.735

### Effect of BPE on colonic metabolite profile in normal and the DSS-treated mice

**Figure 6:**
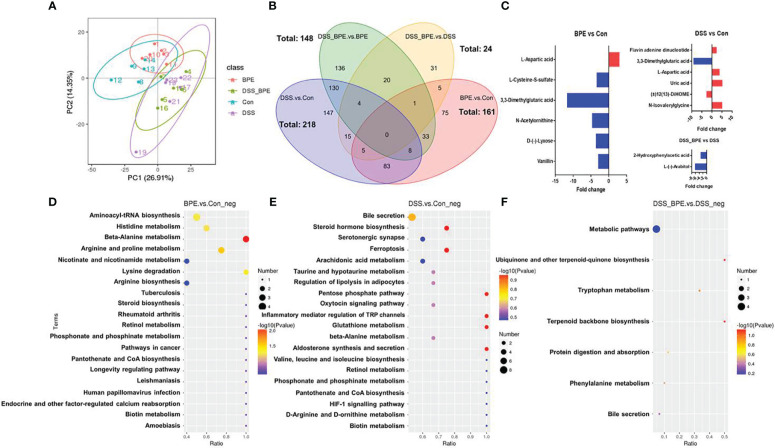
Effect of BPE on colonic metabolomics. **(A)** PCA plot; **(B)** Venn diagram; **(C)** Differentially expressed metabolites in BPE vs Con, DSS vs Con, and DSS-BPE vs. DSS (screening under fold change >2 & VIP >1.5); **(D)** Enriched KEGG pathway in BPE vs Con **(D)**, DSS vs. Con **(E)**; and DSS-BPE vs. DSS **(F)**.

Untargeted metabolomics was used to investigate the effects of BPE in normal and DSS-treated mice (**Figure 6**). In contrast to the Con group, individual BPE treatments led to more clustered colonic metabolites, comprising 161 differentially expressed metabolites (DEMs) such as aspartic acid, cysteine-S-sulfate, 3,3-Dimethylglutaric acid, N-acetylonithine, lyxose, and vanillin. These mainly corresponded to the enriched pathways of alanine, histidine, arginine, and proline metabolism. DSS treatment induced a completely distinct metabolite profile, encompassing 218 DEMs, including flavin adenine dinucleotide, 3,3-Dimethylglutaric acid, aspartic acid, uric acid, 2(13)-Dihome, and N-Isovalerylglycine. These primarily belong to the enriched pathways involving bile secretion, steroid hormone biosynthesis, ferroptosis, and arachidonic acid metabolism. Supplementation with BPE in DSS-treated mice failed to restore the altered metabolite profile, with only 24 DEMs detected, such as 2-Hydroxyphenylacetic acid and arabitol, mainly corresponding to a metabolic pathway.

## Discussion

In this study, flavonoids, sugars, and polyphenols were identified as the main active ingredients, constituting 59% of the ethanol extract of BP leaves. Quinic, geniposidic, chlorogenic, and caffeic acids were the dominant phenolic acids, demonstrating antioxidant and antimicrobial activities. It has been reported that caffeoylquinic acids found in BP leaves extract exhibit strong antioxidant activity, attributed to their double hydroxyl on B-ring (Cao et al., 2020). Furthermore, BP polysaccharides have been shown to display high antibacterial activity against both gram-positive (*B. subtilis* and *S. aureus*) and gram-negative (*E. coli* and *P. aeruginosa*) bacteria by altering the permeability of the cell wall and membrane (Han et al., 2016). Papyriflavonol A (PapA), a prenylated flavonoid isolated from BP root bark, has been found to exhibit broad-spectrum antimicrobial activity against multiple microorganisms and is affected by the disruption of cell membrane integrity (Sohn et al., 2010).

Earlier research involving the supplementation of 150 g/t BP leaf extract for a duration of 42 days, has elucidated improvements in various physiological aspects of weaned piglets, such as growth performance, antioxidant capacity, diarrhea incidence, immune response, and disease resistance (Chen et al., 2020). In the current study, 2% DSS was administered to mice in drinking water to induce inflammation in the colon. Following a one-week period of DSS treatment, symptoms such as weight loss, diarrhea, increased liver and spleen index, disrupted colon architecture, and reduced gene and protein expression related to antioxidant and barrier functions were observed, indicating the successful creation of the model. Similar observations, including decreased body weight, increased diarrhea index, and increased liver and spleen weights, have been reported in DSS-treated mice (Kwon et al., 2021). In addition, this study elucidates the molecular mechanisms underlying the association between DSS-induced colitis and hepatic steatosis. Unexpectedly, co-gavage of 200 mg/kg BPE (equivalent to 4 mg/day/mouse or 20 g) in DSS-treated mice did not restore either the phenotypic change or gene expression related to the antioxidant and barrier functions of the colon. Intriguingly, a single gavage of BPE induced diarrhea after five days of administration and slightly impaired the morphology of the colon, with no other discernible effects. These results contradict those of a study in a weaned piglet model, where even higher levels of BPE were administered without side effects, a discrepancy possibly stemming from variations in the tolerance and utilization capacity of phytochemicals within BPE in different hosts.

In a high-fat diet (HFD)-fed mouse (C57BL6) model, 40 mg/kg of BP root bark extract was intraperitoneally injected daily (0.8 mg/day/mouse as 20 g equivalent), resulting in the downregulation of pro-inflammatory cytokines such as IL-6 and TNF-α in adipose tissue (28). This extracted part of the BP and/or the administration method could be the underlying reasons for the inconsistent results found in our study. In another study, different dosages of polysaccharides extracted from BP leaves (100 mg/kg/d, 200 mg/kg/d, and 400 mg/kg/d) were orally administered to KU mice, showing a dose-dependent mitigation of acetaminophen-induced liver damage and restoration of the downregulated gene expression of Nrf2 and its downstream target genes in the liver, signifying the antioxidant benefits of BP polysaccharides (BPP) (Xu et al., 2022). The specific cause of the differences observed in our study, whether related to the active compounds within the BP leaf extract and/or organ-specific targets, remains ambiguous. The hepatoprotective effect of BPP may be attributed to the metabolic effects of the intestinal microbiota (Xu et al., 2022). Intestinal microorganisms may interact with phytochemicals within BP extracts, exerting direct or indirect effects on the health of the host. In our study, BPE did not shift the intestinal bacterial diversity, but did increase the colonic abundance of *Faecalibaculum* and *Akkermansia*, commensal bacteria known to reduce the risk of gastrointestinal disorders by producing short-chain fatty acids (Lukovac et al., 2014). Similarly, BPE-administered weaned piglets exhibited little change in community richness and diversity but enhanced the abundance of the butyrate-producing Roseburia genus (Lee et al., 2020). Both single treatments with BPE or DSS conspicuously altered the intestinal metabolite profile, although BPE co-treatment did not reverse the changes in DSS-treated mice. BPE may influence amino acid metabolism, such as that of alanine, arginine, and aspartic acid. In the DEM profile, aspartic acid was augmented in both single BPE and DSS treatments. Aspartic acid is one of the most abundant amino acids in the leaves of BP accounting for 23.42% (g/kg DM) (Hao et al., 2021). An overdose of aspartic acid can affect the level of neurotransmitters such as serotonin, and subsequently affect appetite (Czarnecka et al., 2021). Furthermore, excessive aspartate uptake can affect the average daily feed intake, average daily weight gain, intestinal inflammatory response, and gut microbiota in young pigs (Li et al., 2019). Accordingly, it is conjectured that BPE administration may exacerbate intestinal inflammation in a damaged intestinal state. Overall, in contrast to previous studies, our findings do not support the many benefits of BPE in normal and DSS-treated mice; however, certain side effects, such as the induction of diarrhea, were detected. Therefore, more comprehensive research focusing on intestinal health and specific functional molecules is warranted.

## Conclusions

The hybrid *B. papyrifera* (BP) is a species that is widely cultivated and processed into silage for ruminants in China. The abundance of active compounds, coupled with a high protein content, forms the basis of its recognized medicinal and nutritional value. However, literature concerning BP is sparse, with very few studies examining the effects of BP as an unconventional feed ingredient when initially incorporated into dietary feed (Niu et al., 2022). Within the context of the present investigation, we explored the supplementary effects of BPE on both normal and DSS-treated mice with intestinal inflammation. Despite the *in vitro* inhibitory activity of BPE against various pathogens, its efficacy in alleviating DSS-induced intestinal damage was unsatisfactory, a finding consistent regardless of the enrichment of *Faecalibaculum* and *Akkermansia*. It is postulated that the high level of aspartic acid in BPE may have deleterious effects on intestinal health, thus providing a plausible explanation for why BP silage is more commonly utilized after probiotic fermentation.

## Data availability statement

The datasets presented in this study can be found in online repositories. The names of the repository/repositories and accession number(s) can be found below: https://nmdc.cn/resource/genomics/project/detail/NMDC10018471, NMDC10018471.

## Ethics statement

The animal study was approved by the Institute of Subtropical Agriculture, Chinese Academy of Sciences. The study was conducted in accordance with local legislation and institutional requirements.

## Author contributions

XL: Data curation, Methodology, Writing – original draft, Formal Analysis. MR: Data curation, Formal Analysis, Writing – original draft. ZheZ: Formal Analysis, Validation, Visualization, Writing – review & editing. JH: Validation, Writing – original draft. WW: Methodology, Writing – original draft. RW: Validation, Writing – original draft. ZhiZ: Resources, Writing – original draft. KN: Conceptualization, Funding acquisition, Resources, Supervision, Writing – review & editing. XW: Conceptualization, Funding acquisition, Project administration, Writing – review & editing.
