# Supplementary material for: In vitro antibacterial effects of Broussonetia papyrifera leaf extract and its anti-colitis in DSS-treated mice
Source: Front Cell Infect Microbiol. 2023 Oct 16;13:1255127. doi: 10.3389/fcimb.2023.1255127 (PMC10616958; doi:10.3389/fcimb.2023.1255127)
Supplement: Supplementary file 1 [file DataSheet_1.pdf]

Supplementary information

Table S1. Primer information used in the study.

Primer	Direction	Sequence (5'-3')	Product size (bp)	Tm (°C)
GAPDH	Forward	CCTCGTCCCGTAGACAAAATG	133	60
	Reverse	TGAGGTCAATGAAGGGGTCGT		60
TLR4	Forward	GGAACAAACAGCCTGAGACACTT	151	60
	Reverse	CAAGGGATAAGAACGCTGAGAA		60
IL6	Forward	CCCCAATTTCCAATGCTCTCC	141	60
	Reverse	CGCACTAGGTTTGCCGAGTA		60
IL10	Forward	TTTAAGGGTTACTTGGGTTGCC	106	60
	Reverse	AATGCTCCTTGATTTCTGGGC		60
ZO-1	Forward	GGGAAAACCCGAAACTGATG	103	60
	Reverse	GCTGTACTGTGAGGGCAACG		60
Nrf2	Forward	CTGGCTGATACTACCGCTGTTC	208	60
	Reverse	AGGTGGGATTTGAGTCTAAGGAG		60
Cyp1a1	Forward	ACCATGACCGGGAACTGTG	307	60
	Reverse	TGCTGAGGACCAGAAGACCG		60
AHR	Forward	TGGCTTTGTGCTGGTTGTCA	83	60
	Reverse	ACTGCTGAAAGCCCAGGTAATC		60
IL-1β	Forward	GTAATGAAAGACGGCACACCC	181	60
	Reverse	CAGGCTTGTGCTCTGCTTGTG		60
